# Longitudinal Study of Changes in Ammonia, Carbon Dioxide, Humidity and Temperature in Individually Ventilated Cages Housing Female and Male C57BL/6N Mice during Consecutive Cycles of Weekly and Bi-Weekly Cage Changes

**DOI:** 10.3390/ani14182735

**Published:** 2024-09-21

**Authors:** Martina Andersson, Karin Pernold, Niklas Lilja, Rafael Frias-Beneyto, Brun Ulfhake

**Affiliations:** 1 Department of Comparative Medicine, Karolinska Institutet, 171 77 Stockholm, Sweden; martina.andersson@ki.se (M.A.); karin.pernold@ki.se (K.P.); niklas.lilja@ki.se (N.L.); 2 Department of Laboratory Medicine, Karolinska Institutet, 171 77 Stockholm, Sweden; rafael.frias@ki.se; 3 Department of Comparative Medicine, Karolinska University Hospital, 171 76 Solna, Sweden

**Keywords:** Mus musculus, microenvironment, diurnal, toxicity, 3R

## Abstract

**Simple Summary:**

The way animals are housed and cared for in research is vital for both ethical reasons and the quality of research produced. One important aspect of mouse housing is air quality. In this study, we measured levels of two potentially harmful compounds, ammonia and carbon dioxide, as well as humidity and temperature, in mouse cages. This study followed the mice from adulthood (100 days old) to middle age (322 days old) as they gained 35–50% body weight. The mice were divided by sex, and their cages were cleaned either weekly (7 d) or bi-weekly (14 d). Our findings show that air quality remains within the safe levels for younger, lighter mice, but changes throughout the day, with higher levels of carbon dioxide, ammonia, and humidity when the mice are active at night. However, as the mice aged and gained weight, the ammonia and humidity levels increased, potentially reaching harmful levels. Carbon dioxide and temperature were less affected by these changes. Our study suggests that it is important to consider mice’s activity patterns and body mass when determining the frequency of cage cleaning and housing density.

**Abstract:**

Housing conditions are essential for ensuring animal welfare and high-quality research outcomes. In this study, we continuously monitored air quality—specifically ammonia, carbon dioxide, relative humidity, and temperature—in Individually Ventilated Cages (IVCs) housing five female or male C57BL/6N mice. The cages were cleaned either weekly or bi-weekly, and the data were collected as the mice aged from 100 to 348 days. The survival rate remained above 96%, with body weight increasing by 35–52% during the study period. The ammonia levels rose throughout the cleaning cycle, but averaged below 25 ppm. However, in the older, heavier mice with bi-weekly cage cleaning, the ammonia levels reached between 25 and 75 ppm, particularly in the males. While circadian rhythms influenced the ammonia concentration only to a small extent, the carbon dioxide levels varied between 800 and 3000 ppm, increasing by 30–50% at night and by 1000 ppm with body weight. Humidity also correlated primarily with the circadian rhythms (10% higher at night) and, to a lesser extent, with body weight, reaching ≥70% in the middle-aged mice. The temperature variations remained minimal, within a 1 °C range. We conclude that air quality assessments in IVCs should be conducted during animals’ active periods, and both housing density and biomass must be considered to optimise welfare.

## 1. Introduction

Among mammals, small rodents, particularly mice (*Mus musculus*), are commonly used models in biomedical research due to their genetic similarity to humans and their utility in studying a wide range of physiological and pathological processes. Not only for ethical reasons, but also to improve the quality and robustness of research conducted with these animals, various aspects of animal husbandry play a crucial role [1,2,3,4,5]. Among the many factors considered in the husbandry of mice, housing density [6,7,8,9,10,11], the intervals between cage cleanings, and air quality have been studied extensively and in detail [7,9,12,13,14,15,16,17,18,19,20]. The most important measurements in these studies were animal health and air quality, in particular the concentration of ammonia in air (idem). Ammonia is of great concern due to its potentially toxic effect [21,22,23], as ammonia is produced by the enzymatic processing of urea in the urine deposits of mice by endogenous (microbiome) and exogenous (opportunistic) bacterial species (for references, see [24]). Fewer studies have considered other air quality parameters such as carbon dioxide, which is potentially toxic ([15,25] and references therein), and humidity, as high humidity promotes the growth of moulds and bacteria in the cage (see e.g., [13,15,26,27,28]). For simplicity, and probably due to technical challenges, the air quality measurements in the studies cited above were performed as single-point measurements during the day (once a day, every other day, three times a week, etc.), but not around the clock, although we know that mice are nocturnal and that in-cage activities may impact the readings of cage air.

This study was carried out to provide the air concentrations of ammonia and carbon dioxide, air relative humidity, and temperature inside IVCs (Greenline GM500, Tecniplast Spa), each housing five C57BL/6N mice of either sexes around the clock and across successive weekly or bi-weekly cage changes (CC), and moreover when the mice were adult (100 days), and again when they had become middle-aged (322 days). The aims were to reveal if the air parameters changed with the circadian rhythm of rest and activity [29], during the days in between the CC intervals, between the weekly and bi-weekly CC intervals, and as the animals aged [30,31]

## 2. Materials and Methods

### 2.1. Animals and Husbandry

In this study, we used specific pathogen-free (SPF) C57BL/6NCRW mice (*Mus musculus*) of both sexes that were shipped over land from Charles River, Germany, at 6 weeks of age.

**Figure 1:**
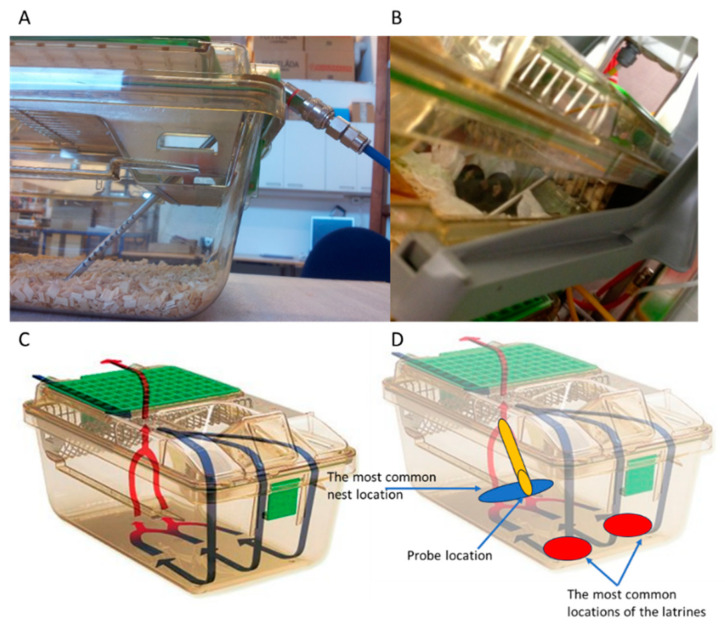
(**A**) This micrograph shows the gas sampling probe with several holes at the distal end. The probe passed through the hole on the rear of the lid, where it was secured by a rubber seal. Individual cables connected the probes of the cages to the sensors located in the adjacent room. A pump sucked air from the cages at a constant rate of 1000 mL h^−1^ per cage. (**B**) The mice tolerated the probe well and often nested near it. (**C**) The airflow dynamics of the IVC GM500 cage, with blue arrows indicating incoming air and red arrows indicating outgoing air. The manufacturer’s recommended ventilation rate is 75 ACH. (**D**) This image shows the IVC, as in C, with the most common locations of the nest(s) (blue area) or latrine(s) (red areas) and the position of the sampling probe (orange profile).

The climate control system of the holding room was set to maintain the temperature at 22 ± 2 °C and the relative humidity (RH) at 40–60% saturation, complying with the legally defined ranges according to Annex 3 of 2010/63/EU. The IVC fans took in air from the holding room to ventilate the cages; the outlet from the cages led to an exhaust pipe (Figure 1). All the cages were individually ventilated with an air handling unit (SmartFlow blower, Tecniplast SpA, Buguggiate, Italy) that delivered HEPA-14 filtered air at 75 air changes per hour (ACH) and negative pressure (−20%, negative inside cage) to all the cages used.

In this study, we used GM500 (Tecniplast SpA, Buguggiate, Italy) cages having a floor size of 501 cm^2^ and overall dimensions of 380 mm (L) × 220 mm (W) × 130 mm (H) (Figure 1). According to the manufacturer’s specification, the disposable air volume for breathing is approximatively 3.5 L. All the cages were prepared with a mixture of 100 g of autoclaved aspen wood shavings (AC) bedding (Tapvei; Paekna, Estonia, 5 × 5 × 1 mm) and 200 g of corn cobs (Bed-o’Cobs ¼” from Datesand, Bredbury, UK) and tissue material for nesting. These requirements were defined to achieve a balance between the efficiency of ventilation based on GM500 airflow dynamics (Figure 1C) and sufficient bedding depth to allow the animals to perform digging activities in accordance with Annex 3 of Directive 2010/63/EU.

All the animals were fed ad libitum with irradiated feed (Standard Diet RM3, Special Diet Service, Route de Saint Bris, France) and had ad libitum access to weakly chlorinated tap water, with a pH of approximately 7.5 and hardness ranging from 6 to 8 dH (degrees of hardness).

### 2.2. Ammonia and Carbon Dioxide Concentration (ppm) Measurements and Assessment of Intra Cage Relative Humidity and Temperature

All the experimental cages were equipped with gas sampling probes that sucked at a constant rate of ~1 L h^−1^, i.e., only a small fraction of the volume of air passing through the IVC per hour. The probes were connected to sensors located in the adjacent room on the opposite side of the wall via cables running through the wall of the recording room. Air that reached the sensors (one set per cage) could be switched between cage air and holding room air by a programmable shutter mechanism. Thus, the sensors were exposed to cage air for only 5 min every hour in period 1 and 5 min every fourth hour in period 2. In the time between the measurements, the sensors were “washed” with room air. The extension of the “washing” time in period 2 was considered necessary due to the rapid wear of ammonia sensors in period 1. The following sensors were used: for ammonia ppm, the electrochemical sensor TR-EC-NH3-1000 (Samon AB, Vellinge, Sweden); for CO_2_ ppm, the TR-IR-(CO_2_) (Samon AB)was used; and for relative humidity and temperature, the Tinytag ultra 2 (Intab, Stenkullen, Sweden) was used. Samon AB was responsible for the customised system and its maintenance, as well as the calibration and replacement of sensors. The sensor results were recorded as csv or text files with date and time stamps. The room air values for ammonia and CO_2_ remained approximately constant during the entire test period, while humidity and temperature in the holding room were allowed to vary within the above-mentioned ranges. Therefore, only the net difference between the room and the cage was used in the statistical testing of changes in humidity and temperature.

### 2.3. Experimental Protocol

On the day of arrival, the animals were weighed and dispensed to the study cages by systematic sampling with a random starting point [26] to form 2 groups of 10 cages with 5 male mice each and 2 groups of 10 cages with 5 female mice each. In addition, several cages with the same number of mice were kept ready as spare cages in case an animal was lost during the experiment. A total of 3 replacement cages were used. A total of 215 animals were used, including 110 females and 105 males. The 4 × 10 cages containing the animals were arranged in period 1 so that the shelf slots were evenly distributed from side to side and top to bottom across the 4 sets of cages. In period 2, after replacing the ammonia sensors, the distribution of the cages across the slots was scrambled to avoid possible bias due to rack placement (for more information, see Supporting Information, Figure S1). The animals were allowed to acclimatise to their new environment, including the gas sampling probe, for several weeks until test recordings were started. After the tests and sensor calibrations, the first period of the experimental recordings started when the animals were 100 days old (4 April) and ended when the animals were 126 days old (on 1 May). The second period began when they were 322 days old (12 November) and ended when they were 348 days old (10 December). A total of 8 cycles of weekly CCs and 4 cycles of bi-weekly CCs were recorded. For the comparison between weekly and bi-weekly CCs, cage days 1–6 were used in the groups with weekly CCs and days 8–13 (i.e., the second week) in the groups with bi-weekly CCs.

The CCs (cage body and grid with the hopper, but not the top lid) were carried out on all the cages on the same day of the week, either once a week or every second week. Neither dirty bedding nor some of the enrichments used were moved from the dirty cage to the clean cage. All the handling of the open cages was conducted in a CC station, which we found reduces the density of airborne particles in the holding room [29] (also see Supporting Materials, Figure S2).

### 2.4. Data Processing and Analyses

Due to the wear and tear of ammonia electrodes and to prevent the data from becoming too numerous, the experiment was divided into two periods (see above). Period 1 was when the animals were from 100 to 126 days old, and the second period was when the animals were from 322 to 348 days old. The data came either from observation protocols, such as the weighing of body mass, the locations of the nest and latrines, and ill-health or death, as well as from digital outputs from the sensors. Most of the data are as time series and were organised by date (day; 24 h) and time stamp or by daytime (lights on; 06:00–18:00) and nighttime (lights off; 18:00–06:00), and weekly and bi-weekly CC intervals, where the CC period ranges either from day 1 to 7 or from day 1 to 14. The day of CC was excluded from analyses because the cages were taken off the rack and also because the CC procedure created a rather noisy environment. Primary processing was performed using spreadsheets, and the data were stored in CSV files for further statistical analyses using R (v. R×64 4.1.1).

It should be noted that since the animals were group-housed, all the sensor data presented in this study apply to all the mice in the cages.

### 2.5. Statistical Analysis

To test the differences across sexes, ages, consecutive CC cycles, CC intervals, days and from daytime to nighttime, and days in the cycles, we performed repeated measures analysis using the rank-based analysis of variance-type statistics (ATSs), as implemented in the nparLD R Software package v. 2.1 [32,33]. We chose a non-parametric test instead of repeated measures ANOVA because normality assumptions were violated in some cases. We considered the cages as the subjects; time, event, and observation orders as the within-subject factors (“sub-plot” repeated factors); and sex and CC intervals as the between-subject factors (“whole-plot” factors). According to the authors’ terminology [34], we used either F2-LD-F1 (sex and CC intervals as the whole-plot factors and observation order as the repeated factor) or F1-LD-F2 (sex or CC intervals as the whole-plot factor and event and observation orders as the repeated ones), and F1-LD-F1 or LD-F1 when only the observation order was used as the sub-plot factor. The statistical analysis of time series with nparLD was based on the rank order of the observed data, with the relative effect size (*p*_s_) as the effect size measure [33]. Instead of the mean difference in parametric tests being between observations, the rank order was used to assess the probability that two sets of observations differ (*p*_s_ = 0.5 means that there is no difference in rank order) and with longitudinal data if the relative effect size varied across time series within the sets of observations.

In the plots and graphs, the data are given as mean ±SD or SEM, or as mean and the 95% confidence interval (CI). A probability (*p*) of ≤5% (0.05) was considered significant; in the following text, the calculated *p* value is indicated.

## 3. Results

### 3.1. Female and Male Mice

**Figure 2:**
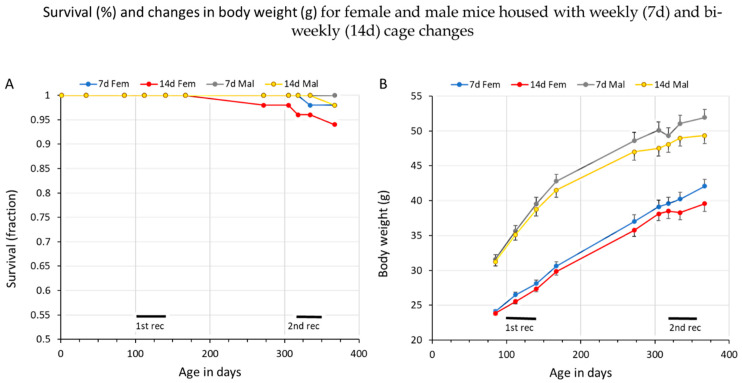
(**A**) This shows the survival of the mice used in this study for each sex and housing condition. The colour code at the top of panel B indicates the housing conditions. The recording periods are marked with black lines. (**B**) This shows the average and SEM of body weight (in grams) for the female and male mice housed with weekly (7 d) and bi-weekly (14 d) cage changes, respectively. The colour code at the top of panel A indicates sex and the different housing conditionsThe periods of the first and second recordings are marked with black lines.

The survival rate of the animals at the end of this study, which lasted almost a year, was >96% (Figure 2A). Two females from the cohort with bi-weekly CCs and one male from a cage with weekly CCs were lost and replaced. Shortly after the end of recording, two more animals were found dead (Figure 2A). All these incidents were classified by the responsible veterinarians as sudden deaths of unknown cause. One female animal was removed early prior to measurements due to eye problems.

The total body weight increased as expected in both the sexes during the experimental period (Figure 2B), and the observed growth rates compare well with the growth curves published for the C57BL/6N strain. Between the ages of 100 days (period 1) and 322 days (period 2), the female mice increased from ~25 g to ~38 g (+52%), while the males increased from ~34 g to ~48 g (+35%). Thus, the difference in average intra-cage biomass between the female and male mice decreased slightly between the two recording periods, from 136% (males vs. females) at 100 days of age to 126% at 322 days of age.

### 3.2. Location of Nests and Latrines

**Figure 3:**
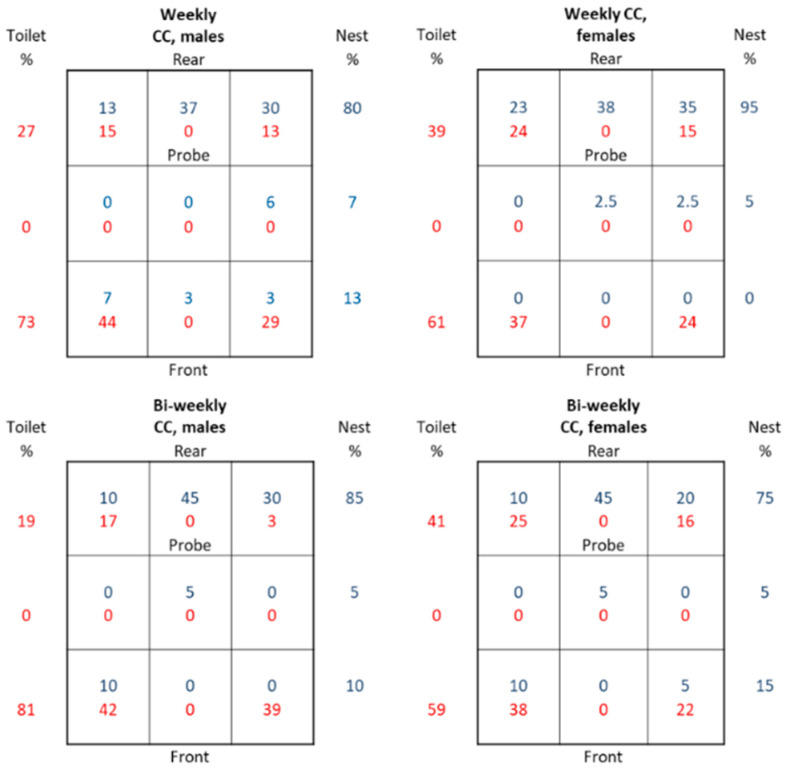
Four panels showing the location of the latrines (toilets) and nests in the cages with the female and male mice and weekly or bi-weekly CCs as indicated. The percentage of latrine (red) and nest (blue) locations noted is shown for each of the nine areas into which the floor was divided. The row totals are shown on the left and right, respectively. The location of the sampling probe is indicated in each panel.

We noted the placement of the nests and latrine(s) during welfare checks and the CCs (Figure 3). For simplicity, we divided the floor of the cage into nine sections with three rows, with a row in the front, one in the rear, and one in between (Figure 3). Both the female and male mice preferred to nest in the rear row. It was preferred to have a nest in close proximity to the gas sampling probe (37–45%) (see Figure 1 and Figure 3). Although this study is not a controlled exposure study, the preferred location of the nest, where the animals are still for longer periods of time, suggests that our environmental readings are a good proxy for the exposure of animals.

In contrast, the preferred location of the latrine(s) was in the front row, especially among the male mice (73% and 81%). Somewhat unexpectedly, the females had latrines in the back row in ~40% of cases, i.e., close to the nest in many cases. A latrine was not observed in the floor areas of the middle row (Figure 3).

### 3.3. Intra-Cage Ammonia Levels across Day and Night and Cage Change Cycle

**Figure 4:**
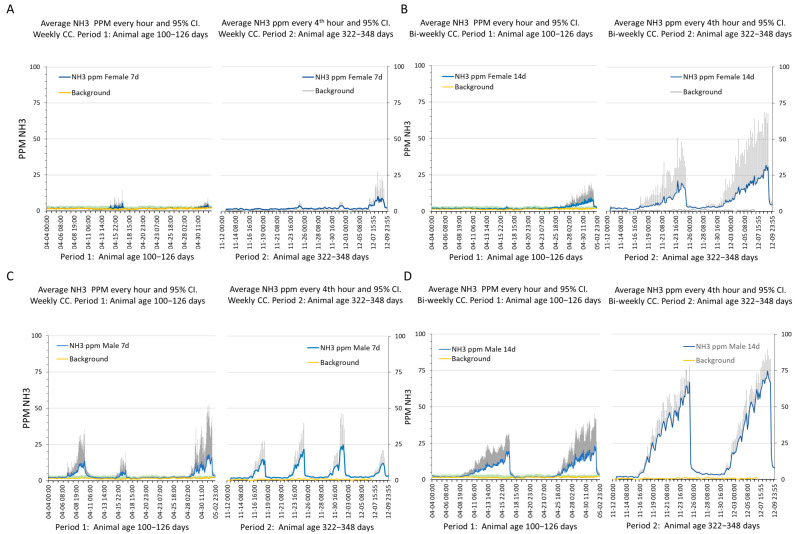
(**A**–**D**) Plots of average (blue line) and 95% CI (light grey error bar) of NH3 ppm every hour (left side of each panel) or every fourth hour (right side of each panel) during consecutive cycles of weekly (**A**,**C**) and bi-weekly (**B**,**D**) CCs. A-B panels show female mice data, while (**C**,**D**) are panels showing male mice data. Period 1 (age 100–126 days) and period 2 (age 322–348 days) are marked (on the abscissa) to reflect animal age and experimental phase. Date and time format on the abscissa is month:day:h:min.

The ammonia content (ppm) of the gas samples was measured once per hour during the first recording period and every 4 h during the second period (see Section 2). On average, the ammonia content was ≤7 ppm across all the days during both period 1 and period 2 in the cages with weekly CCs. As can be seen in Figure 4, the ammonia ppm increased during the CC interval, but both the average and peak values remained below 25 and 50 ppm, respectively, in period 1 for both the sexes and both the CC intervals (Figure 4A–D). In period 2, when the animals were older (322–348 days c.f. 100–126 days) and had a higher body weight (see above), the cages with the female and male mice with weekly CCs still showed an ammonia ppm that did not exceed 50 ppm. In the cages with bi-weekly CCs, the females reached levels of 25 and 50 ppm or more towards the end of the CC cycle (Figure 4B). The corresponding cages with males reached over 50 ppm and up to 75 ppm in the second week of the CC cycle (Figure 4D). Thus, the data indicate that the day of the CC cycle, the frequency of CCs, and age (representing the order of CC cycles), with an increase in body weight as well as sex influence the ammonia level in the cage.

Statistical analyses of the longitudinal data [series] within the sexes showed the significant influence of the day in the CC cycle (females *p* = 4.8 × 10^−13^; males *p* = 4.6 × 10^−20^), the CC interval (females *p* = 1.3 × 10^−3^; males *p* = 2 × 10^−6^), and age (females *p* = 1 × 10^−4^; males *p* = 4.8 × 10^−6^) (whole model NH3ppm~day-in-CCcycle*CCinterval*age; females *p* = 5 × 10^−3^; males *p* = 2 × 10^−4^). Using the same approach, we analysed the effects of day and night (i.e., circadian rhythm) and found a discrete, but consistent difference with significantly higher values at nighttime in the cages with the males (*p* = 7.4 × 10^−16^); in the cages with the females this covariation was smaller and only weakly significant (*p* = 3 × 10^−2^) (see Supporting Materials, Figure S3). The effect of sex on ammonia concentration was significant in both the cages with weekly (*p* = 4 × 10^−2^) and bi-weekly (weekly *p* = 1 × 10^−2^) CCs, but the overall model was only significant for the cages with bi-weekly CCs (NH3 ppm~sex*age*day in cycle, *p* = 2 × 10^−2^).

### 3.4. Intra-Cage Carbon Dioxide Levels

**Figure 5:**
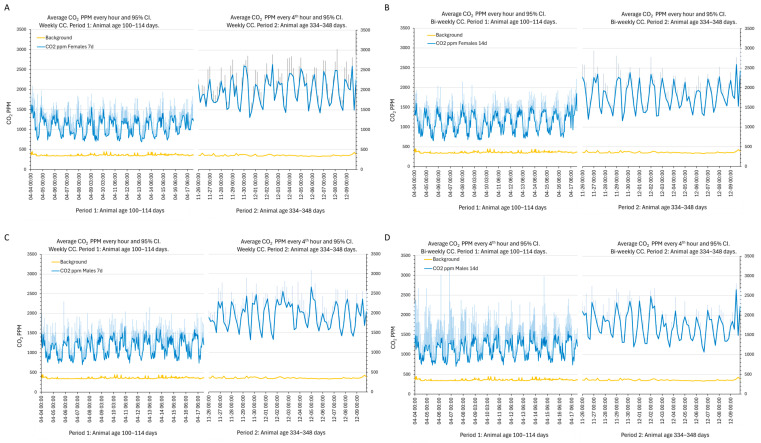
(**A**–**D**) The plots of the average (blue line) and 95% CI (light blue error bar) of CO_2_ ppm every hour (left side of each panel) or every fourth hour (right side of each panel) during the first and last two weeks of recordings to resolve the circadian pattern of variations in the cages with female (**A**,**B**) and male (**C**,**D**) mice having weekly (panels to the left) and bi-weekly (panels to the right) CCs. Period 1 (100–114 days) and period 2 (334–348 days) are indicated on the abscissa. Date and time format on the abscissa is month:day:h:min. The ordinates show ppm CO_2_. The background levels of CO_2_ ppm are indicated by orange lines in each panel and were typically 350–400 ppm.

The carbon dioxide content (ppm) varied with the phase of the circadian cycle (±500–1000 ppm; females *p* = 2.6 × 10^−73^, males *p* = 4.3 × 10^−48^) and the age of the animals (100–126 days vs. 322–348 days, ~1000 ppm; females *p* = 8.9 × 10^−39^, males *p* = 1 × 10^−29^) (Figure 5 and Supporting Materials Figure S4), but not with the day in the cycle or the CC interval or sex (idem).

### 3.5. Intra-Cage Humidity (%) and Temperature (°C)

**Figure 6:**
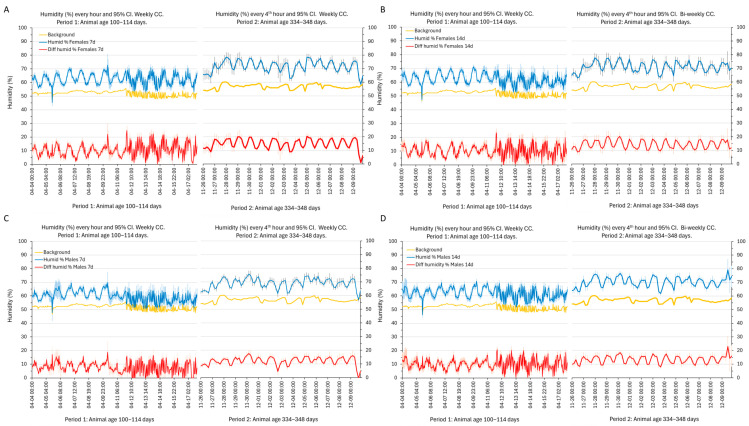
(**A**–**D**) The plots of the average (blue line) and 95% CI (light blue error bar) of relative humidity (%; ordinates) at every hour (left side of each panel) or every fourth hour (right side of each panel) during the first and last two weeks of the recordings to resolve the circadian pattern of variations in cages humidity with female (**A**,**B**) and male (**C**,**D**) mice having weekly (panels to the left) and bi-weekly (panels to the right) CCs. Period 1 (100–114 days) and period 2 (334–348 days) are indicated on the abscissa. Date and time format on the abscissa is month:day:h:min.The background humidity is indicated by an orange line in each panel. The average difference between the humidity in the cage and the background humidity is indicated by a red line (lower trace in each panel) together with the 95% CI. The high-frequency fluctuations in the humidity values (background values and measurements inside the cage) in the latter part of period 1 were due to a technical error.

During period 1, the humidity in the cage was, on average, 7–11% higher than that in the holding room, and during the second period, it was between 11 and 13% higher. The circadian rhythm of rest and activity had a major influence (average difference day vs. night ~10% saturation), with higher humidity at night (females *p* = 3.5 × 10^−98^, males *p* = 2.5 × 10^−49^) (Figure 6; also see Supporting Materials Figure S5). Humidity also varied with age (and thus body weight) (females *p* = 1.3 × 10^−74^, males *p* = 4.7 × 10^−98^), with higher mean values in the cages with mice of older ages, but the circadian fluctuations in humidity had smaller amplitude (~5%, Figure 6; also see Figure S5 in the Supporting Materials). There was a smaller effect of sex on cage humidity in the cages with weekly CCs (*p* = 4.3 × 10^−8^; Figure 6, also see Supporting Materials, Figure S5), but not with bi-weekly CCs.

**Figure 7:**
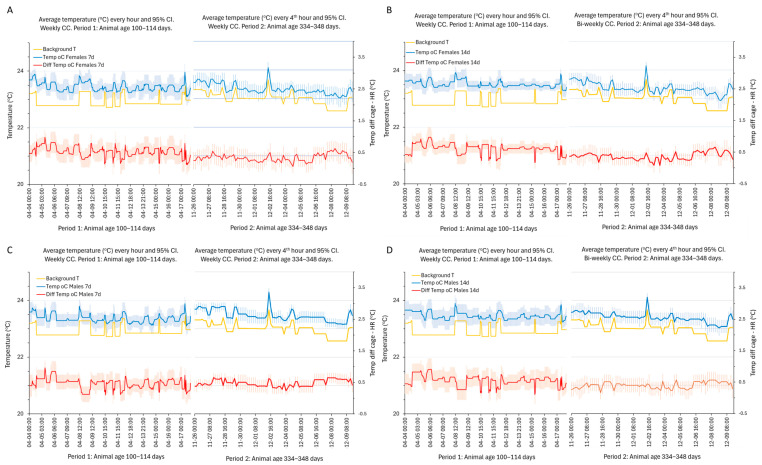
(**A**–**D**) The plots of the average (blue line) and 95% CI (light blue error bar) of temperature (°C; ordinates) at every hour (left side of each panel) or every fourth hour (right side of each panel) during the first and last two weeks of the recordings to resolve the circadian pattern of variations in cages temperature in cages with female (**A**,**B**) and male (**C**,**D**) mice having weekly (panels to the left) and bi-weekly (panels to the right) CCs. Period 1 (100–114 days) and period 2 (334–348 days) are indicated on the abscissa. Format of date and time on the abscissa is month:day:h:min. The background temperature is shown as an orange line in each panel. The average difference between the temperature in the cage and the background temperature is indicated by a red line together with the 95% CI. The left ordinates show humidity in %, and the right ordinates show the temperature difference between the cage and the background.

The difference between the intra-cage temperature and the background was typically small, around 0.5 °C during period 1 and slightly smaller during period 2 (Figure 7). The amplitude of variation across the days and nights and over time stayed within 1 °C (idem).

Modelling intra-cage humidity as a function of the day in the CC cycle, the CC interval, and the cycle number (proxy for the animals’ age) failed to reveal any statistically significant differences.

### 3.6. Impact by Disrupted Cage Ventilation

**Figure 8:**
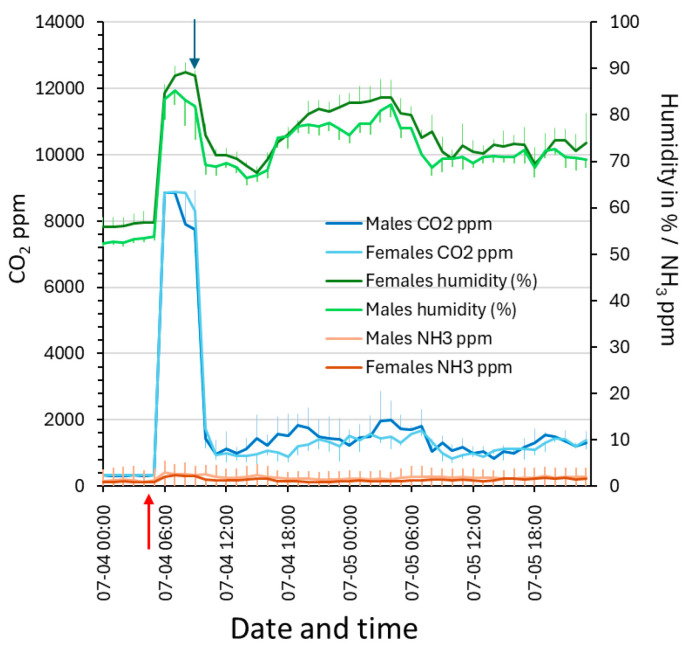
This diagram shows the effects of interrupted cage ventilation (red arrow) on carbon dioxide ppm (left ordinate), humidity, and ammonia ppm in the cages (on the right ordinate). When ventilation was resumed (blue arrow), the sensor readings returned to normal values. Format of date and time on the abscissa is month:day:h:min.

In a set of three male and three female cages, aeration was interrupted for 3 h, and then resumed at 75 ACH (Figure 8). Interrupted aeration caused humidity in the cage to rise rapidly to ~90% and CO_2_ ppm to reach the equilibrium gradient of the cage filter (at ~9000 ppm or 0.9%), while both the ammonia levels (doubled) and temperature (+1 °C) rose more slowly. After restarting aeration, all the parameters returned to the normal levels at 75 ACH within ~4 h (Figure 8).

## 4. Discussion

### 4.1. General Comments

The data presented herein provide a unique insight into how air quality is affected by the circadian rhythm of rest and activity in the home cage [35] (reviewed in [36]), as well as the effects of aging and weight gain [30,31] associated with aging well into middle age in mice. Importantly, during this study, which spanned 246 days, there were no reports of disease, and only three animals were lost by 348 days of age, which compares favourably or even better than many other studies of aging in this mouse strain. The increase in body weight was in line with the data published by the breeder.

The C57BL/6N strain commonly used in biomedical research may differ from other strains like CD1 in metabolic rate, activity levels, and ammonia production, e.g., [34]. These variations could affect air quality. This study’s limitation is that only one strain and one housing density were tested [6,7], which may not reflect the outcomes of the other strains. The studies specific to laboratory mice, e.g., [37], have indicated that the ammonia exposure thresholds and impacts may vary between mouse strains. As discussed elsewhere [16,24,38], the use of human exposure standards for ammonia, the 25 ppm average exposure limit, may not be directly applicable to laboratory mice, and moreover direct comparison across studies on mice is complicated by the use of different techniques to measure the in-cage NH_3_ concentration. The absence of established ammonia exposure limits for mice remains a limitation, and further research is needed to determine the most appropriate safety guidelines for laboratory rodents (also see below).

Furthermore, we did not directly measure the activity levels in the cage, such as feeding or drinking behaviour, or the variations in bedding materials [17,39]. Instead, the impact of the circadian rhythm on air quality was inferred from known nocturnal behaviour patterns of mice, as described in the previous studies [31,35,40].

### 4.2. The Influence of Day of CC Cycle and Length of CC Cycle

The change in air quality during the CC cycle and the length of the CC interval mainly affected airborne ammonia formation, which is consistent with the previous studies [13,16,17,27,28,39]. As discussed elsewhere, the rather wide range of ammonia concentrations reported in the literature (e.g., [8,41]) is probably due to differences in the measurement techniques and the mouse strains used [42].

Following the exposure limits for humans, the IVCs housing five male or female C57BL6/N mice in adulthood (100 days) during bi-weekly CCs were below the upper exposure limit for ammonia. With increasing age and associated increases in body weight (>180 g/cage), metabolic and physiological changes, such as increased nitrogen metabolism and greater overall metabolic activity, lead to a higher production of waste products as urea. In-cage ammonia is primarily produced from the urea in urine. As animals grow older and gain body mass, the volume of urine excreted increases, providing more substrates for the microbial breakdown of urea into ammonia within the cage environment. This results in a higher concentration of ammonia, particularly towards the end of the CC cycle, where it can exceed the upper exposure limits of 25 (average) and 50 (peak) ppm. The rationale for adopting the safety guidelines for ammonia exposure in humans (≤25 ppm average exposure over a working day and a peak exposure of ≤50 ppm) in laboratory rodents remains unclear, as discussed in detail in [8,13,24], but it is an open question that deserves further attention, as there are gross discrepancies between the reported ammonia ppm levels and the histopathologic findings in the upper respiratory tract (e.g., [8,13,16,17,24,27]). One possible explanation for this discrepancy is that this study and the studies cited above are not considered controlled exposure studies, as we only infer actual exposure from the measured values of cage air samples (see discussion in [8,24]).

The reason for extending the CC interval from one to two or more weeks is not mainly to reduce housing costs, but because of the scientific argument that CC fundamentally disrupts the rhythm of daily activity in a cage, including the disruption of sleep patterns (see the discussions in [35,40,43,44]).

In line with the observation that the ammonia in-cage load increases as the animals aged and became heavier, the level of in-cage carbon dioxide also increased, reflecting the increase in biomass with age (~1000 ppm between 120 and 322 days of age with the strain and housing density used here). Also, in-cage humidity was 5–10% higher in the cages with bi-weekly CCs, but did not show an association with the day of the CC cycle.

### 4.3. The Impact of the Circadian Rhythm

A novel observation made here is that the concentrations of ammonia and carbon dioxide in the air, as well as the humidity and air temperature in the IVCs, showed a circadian variation that was probably caused by the activity and resting patterns of the mice in the cages [31,35,40]. The circadian oscillations had amplitudes of >10% when the animals were adults, but were smaller (5–10%) when the mice were middle-aged. One explanation for the lower amplitude in the middle-aged mice could be that, as shown elsewhere, older mice are less active in their home cage than younger adult mice [30,45]. The amplitude of carbon dioxide variation across the circadian cycle was ~500 ppm in the adults (100–126 days old) and ~1000 ppm in the cages with the middle-aged mice (322–348 days old), a change that is mainly driven by the increase in body mass since the level of activity usually decreases with advancing age for the C57BL/6 strain.

We also found a variation between day and night in the time series of ammonia concentration and temperature, but the effects were quite discrete.

### 4.4. The Influence on Air Quality of Sex, Age and Body Weight Gain

The sex difference in the build-up of ammonia concentrations during a cage cycle in mice is well known (see the discussion and references in [24]) and is related to the importance of urine markings in hierarchical play within groups of mice housed in a cage. The difference in body mass between sexes is also important [24], as nitrogen metabolism is related to organismal mass [22], and as shown here, also increases significantly in females from adulthood to middle age (+52% in female mice). The observed difference in body mass between the sexes appears to be balanced by the sex difference in home cage activity [3,24], as we found very little difference in the carbon dioxide concentration and humidity between the sexes. In line with the observation that the in-cage load of ammonia increased as the animals aged and became heavier, the level of in-cage carbon dioxide also increased by ~1000 ppm between 120 and 322 days of age, reflecting the increase in biomass with age (with the strain and housing density used here). However, the carbon dioxide level stayed below 0.35% of cage air and a concentration that should not pose a welfare problem.

Humidity was ~10% higher in the cages than that in the holding room when the animals were adults and ~15% higher when the animals were middle-aged, reaching levels between 65 and 75% saturation. Although the holding room had a humidity saturation of ≤60%, the contribution of the mice in the cage resulted in excessive humidity, which increased the risk of unwarranted mould growth between cage cleanings. Thus, one lesson from this study is that humidity in the holding room (air supply for the type of ventilators used in this study) should probably not exceed 45% to be on the safe side.

## 5. Conclusions

This study shows that for the C57BL/6N mice housed at a density of five per GM500 cage subjected to weekly and bi-weekly cage changes, there was a significant deterioration in air quality as the animals aged and gained body mass, posing a risk to animal welfare. Up to an age of 3–4 months, or a combined biomass of <180 g, the bi-weekly CCs were not associated with air quality problems. In addition to elevated ammonia, the humidity levels were also higher in the bi-weekly changed cages, contributing to a suboptimal environment. To mitigate these negative effects, we recommend reducing the humidity of the air supply, decreasing the housing density in long-term studies of mice, or implementing weekly cage changes to maintain an acceptable air quality. The careful consideration of cage cleaning intervals and housing density is essential to ensure the welfare of laboratory mice, especially in aging populations, where their biomass increases over time.

## Data Availability

Upon request to the CA, the raw data and the meta data will be provided.

## Supplementary Materials

The following supporting information can be downloaded at: https://www.mdpi.com/article/10.3390/ani14182735/s1, Figure S1: The diagram shows the placement of cages with female and male mice, which are changed weekly or bi-weekly. The upper diagram shows the placement during period 1 and the lower diagram shows the placement during period 2. The colour coding key is at the bottom. Figure S2: Measurement of the density of airborne particles of different sizes (large = P1, small PM10 and in between PM4 and PM2.5) in the holding room 30 min before a cage change, during a cage change and 30 min after a cage change. Mean values and SD are shown. The measurements of the particle density in the air of the enclosure were carried out with the Met One Instrument Model 831 and the values are given in µg/m^3^ air. The size groups PM10, PM4, PM2.5 and PM1 refer to particle sizes ≤10, ≤4, ≤2.5, ≤1 µm. Figure S3: Relative effect size (ordinate in all panels) of the day in the CC cycle, the CC interval and the number of the CC cycle (representing the age of the animals) on the daily ammonia content in the cage (ppm) (4 panels on the left). The panels on the right show the effect size of the circadian rhythm (day and night), the CC interval and the age of the animals on the ammonia concentration in the cage. Figure S4: Relative effect size (ordinate in all panels) of the day in the CC cycle, the CC interval and the number of the CC cycle (representing the age of the animals) on the daily carbon dioxide content (ppm) within the cage (4 panels on the left). The panels on the right show the effect size of the circadian rhythm (day and night), the CC interval and the age of the animals on the carbon dioxide concentration inside the cage. Figure S5: Relative effect size (ordinate in all panels) of the day in the CC cycle, the CC interval and the number of the CC cycle (representing the age of the animals) on the daily humidity in the cage (% saturation; the 4 panels on the left). The panels on the right show the effect size of the circadian rhythm (day and night), the CC interval and the animal age on the humidity in the cage.
