# Supplementary material for: Longitudinal Study of Changes in Ammonia, Carbon Dioxide, Humidity and Temperature in Individually Ventilated Cages Housing Female and Male C57BL/6N Mice during Consecutive Cycles of Weekly and Bi-Weekly Cage Changes
Source: Animals (Basel). 2024 Sep 21;14(18):2735. doi: 10.3390/ani14182735 (PMC11428529; doi:10.3390/ani14182735)
Supplement: Supplementary file 1 [file animals-14-02735-s001.zip › animals-3200819-supplementary.pdf]

Supporting Materials to

A longitudinal study of changes in ammonia ppm, carbon dioxide ppm, humidity, and temperature in IVC cages housing female and male C57BL/6N mice during consecutive cycles of weekly and bi-weekly cage-change

by M. Andersson, K. Pernold, N. Lilja, R. Benito Frias, and B. Ulfhake

Figure S1

Period 1

Empty	Empty	Empty	Empty	Empty	Empty	Empty	Empty
29	NA	24	Room	NA	53	48	Empty
10	NA	3	13	NA	34	66	NA
1	NA	15	21	NA	64	43	NA
28	NA	9	47	NA	50	38	NA
46	NA	63	36	NA	16	22	57
56	NA	45	NA	NA	19	20	55
58	NA	62	NA	NA	23	5	8
49	NA	61	NA	NA	26	30	27
Empty	Empty	Empty	Empty	Empty	Empty	Empty	Empty

Period 2

Empty	Empty	Empty	Empty	Empty	Empty	Empty	Empty
26	NA	46	Room	16	63	36	Tom
NA	56	NA	NA	50	38	49	NA
66	NA	62	19	NA	NA	47	NA
28	NA	48	5	NA	24	3	NA
53	58	22	NA	27	30	9	13
23	64	8	20	10	NA	NA	NA
NA	NA	NA	45	1	NA	NA	NA
57	55	15	29	61	21	NA	NA
34	NA	43	NA	NA	NA	NA	NA

Bi-weekly CC, Females
Bi-weekly CC Males
Weekly CC Females
Weekly CC Males

Figure S1 The diagram shows the placement of cages with female and male mice, which are changed weekly or bi-weekly. The upper diagram shows the placement during period 1 and the lower diagram shows the placement during period 2. The colour coding key is at the bottom.

Figure S2

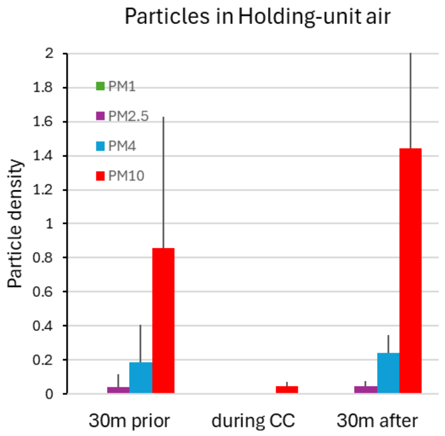

Figure S2 Measurement of the density of airborne particles of different sizes (large=P1, small PM10 and in between PM4 and PM2.5) in the holding room 30 minutes before a cage change, during a cage change and 30 minutes after a cage change. Mean values and SD are shown.
The measurements of the particle density in the air of the enclosure were carried out with the Met One Instrument Model 831 and the values are given in µg/m^3 air. The size groups PM10, PM4, PM2.5 and PM1 refer to particle sizes ≤10, ≤4, ≤2.5, ≤1 µm.

Figure S3

Intra cage ammonia levels

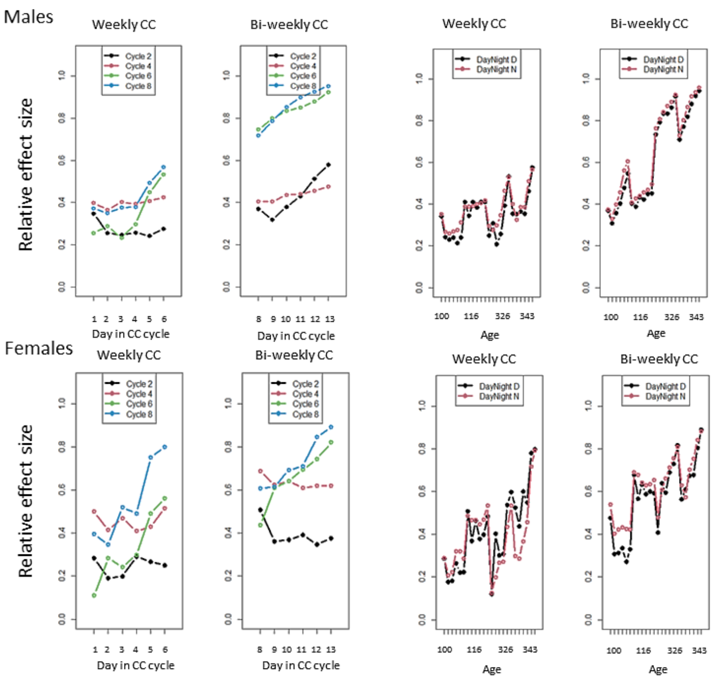

Figure S3 Relative effect size (ordinate in all panels) of the day in the CC cycle, the CC interval and the number of the CC cycle (representing the age of the animals) on the daily ammonia content in the cage (ppm) (4 panels on the left). The panels on the right show the effect size of the circadian rhythm (day and night), the CC interval and the age of the animals on the ammonia concentration in the cage.

Figure S4

Intra cage carbon dioxide levels

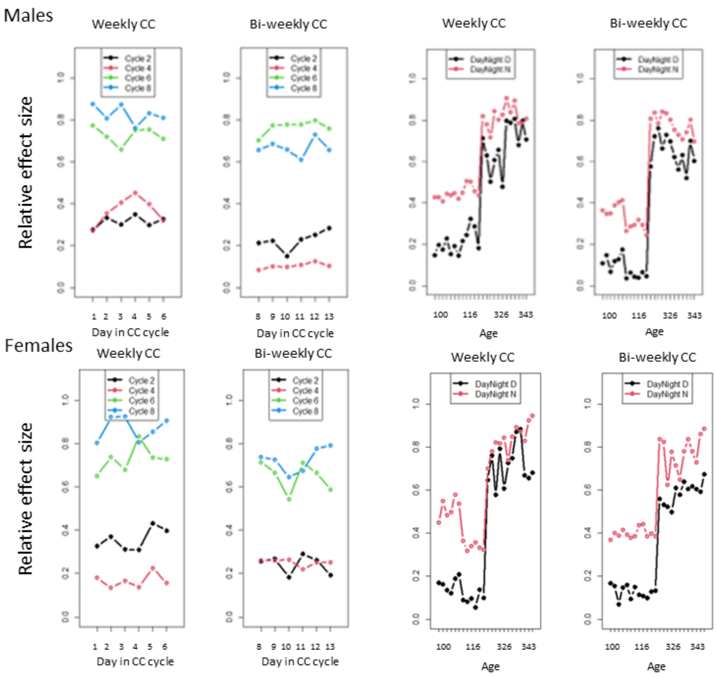

Figure S4 Relative effect size (ordinate in all panels) of the day in the CC cycle, the CC interval and the number of the CC cycle (representing the age of the animals) on the daily carbon dioxide content (ppm) within the cage (4 panels on the left). The panels on the right show the effect size of the circadian rhythm (day and night), the CC interval and the age of the animals on the carbon dioxide concentration inside the cage.

Figure S5

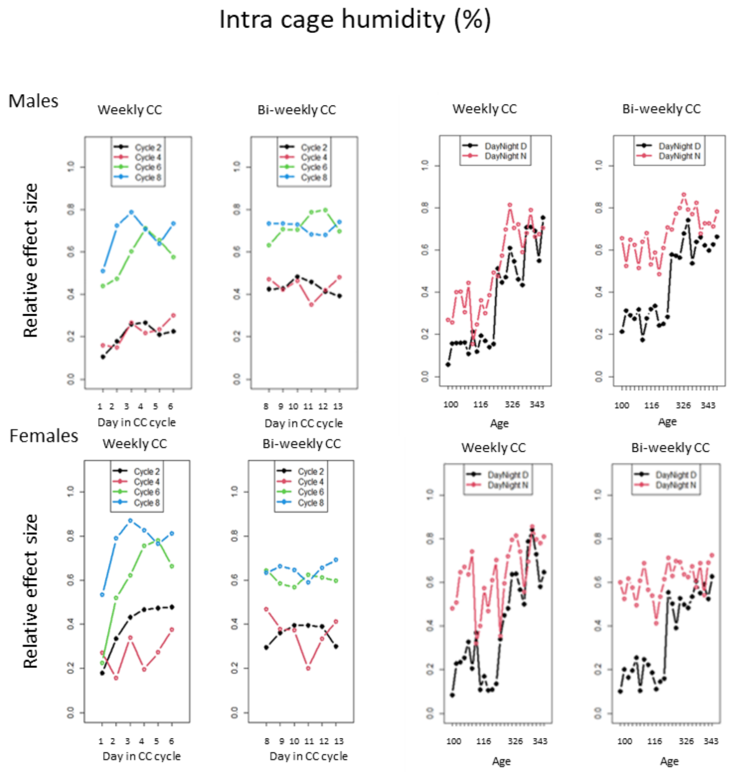

Figure S5 Relative effect size (ordinate in all panels) of the day in the CC cycle, the CC interval and the number of the CC cycle (representing the age of the animals) on the daily humidity in the cage (% saturation; the 4 panels on the left). The panels on the right show the effect size of the circadian rhythm (day and night), the CC interval and the animal age on the humidity in the cage.
